# Forest Landscape Effects on Dispersal of Spruce Budworm *Choristoneura fumiferana* (Clemens, 1865) (Lepidoptera, Tortricidae) and Forest Tent Caterpillar *Malacosoma disstria* Hübner, 1820 (Lepidoptera, Lasiocampidae) Female Moths in Alberta, Canada

**DOI:** 10.3390/insects13111013

**Published:** 2022-11-02

**Authors:** Barry J. Cooke

**Affiliations:** Canadian Forest Service, Great Lakes Forestry Centre, 1219 Queen Street East, Sault Ste. Marie, ON P6A 2E5, Canada; barry.cooke@nrcan-rncan.gc.ca

**Keywords:** dispersal, outbreak cycles, synchronization, forest landscape fragmentation, spruce budworm, forest tent caterpillar

## Abstract

**Simple Summary:**

Movement patterns of adult female moths in two major forest insect pest species are shown to be governed by two key factors: population density and host forest cover. As populations rise over the course of an outbreak cycle, defoliation rates rise, and this promotes female movement away from defoliated forest stands, into areas with higher forest cover. This suggests mass dispersal at the peak of an outbreak may be adaptive.

**Abstract:**

Leaf-rollers and tent caterpillars, the families Torticidae and Lasiocampidae, represent a significant component of the Lepidoptera, and are well-represented in the forest insect pest literature of North America. Two species in particular—spruce budworm (*Choristoneura fumiferana* (Clem.)) and forest tent caterpillar (*Malacosoma disstria* Hbn.)—are the most significant pests of the Pinaceae and Salicacae, respectively, in the boreal forest of Canada, each exhibiting periodic outbreaks of tremendous extent. Dispersal is thought to play a critical role in the triggering of population eruptions and in the synchronization of outbreak cycling, but formal studies of dispersal, in particular studies of long-range dispersal by egg-bearing adult females, are rare. Here, it is shown in two independent studies that adult females of both species tend to disperse away from sparse or defoliated forest, and toward intact or undefoliated forest, suggesting that long-range dispersal during an outbreak peak is adaptive to the species and an important factor in their population dynamics, and hence their evolutionary biology.

## 1. Introduction

Dispersal has been theorized to play a critical role in animal population ecology, from the alleviation of competition [1], to the survival of species [2], to the distribution of genetic diversity [3], from glacial biogeography [4] to the spread of invasive organisms [5,6]. Dispersal is so intimately associated with density-dependent population regulation that it is thought to be adaptive [7]. Dispersal is challenging to study; but the more it is studied, the more we learn that it plays a wide set of roles in animal population ecology and evolution, from speciation [8] to hybridization introgression [9] to the early success of invasive species establishment and spread [10] to the colonization of new resource patches and metapopulation persistence [11] to the rescue of endangered populations [12] to the alleviation of inbreeding depression [13] to the escape of predation, parasitism and disease [14].

In theory, the effects of dispersal in a coupled predatory-prey system will depend on the species’ growth and interaction dynamics [15]. Early empirical evidence that insect dispersal is a critical process whose success determines whether predatory-prey population cycling is stable or unstable came from Huffaker’s famous experiment with orange mites [16]. Successful dispersal of prey at the bottom of a predator-induced population cycle is what allowed prey to escape predator, thus enabling a subsequent prey population cycle. Only by limiting mite dispersal through the use of bridges and barriers could Huffaker prevent both over-colonization of food resources and over-predation, and thereby force predator and prey to cycle persistently. If prey and predator were allowed to disperse freely, both components went extinct before a full population cycle would be completed.

Studies of insect dispersal at the landscape scale are rare, and yet these are the scales that matter most to large-scale disturbances, such as forest insect outbreaks, the focal topic of this paper. Long-range dispersal through winged flight during the adult life stages, by egg-bearing females in particular, is thought to play a critical role in the spread of both native [17] and introduced forest insect species [18], and in the amplification [19] and synchronization [20] of forest Lepidopteran population cycles. A review of any pest risk analysis will reveal that one of the major uncertainties in evaluating pest risk is the persistent knowledge gap surrounding the factors that promote and govern long-distance dispersal [21]. Knowing that a forest pest such as spruce budworm, *Choristoneura fumiferana* (Clem.), occasionally engages in long-range migratory flights [22] is only the first step toward understanding the dispersal process in enough ecophysiological detail that long-range flight trajectories, from takeoff to landing, can be accurately simulated year after year [23,24].

In contrast to spruce budworm, forest tent caterpillar, *Malacosoma disstria* Hbn., has been characterized as a “weak flier” [25], and yet it performs well on flight mills in the laboratory [26], and it has been observed dispersing several hundred kilometres on occasion [27]. Forest tent caterpillar is assumed to have little difficulty dispersing among forest patches relative to their natural enemies [28], thus resulting in prolonged outbreaks in fragmented forests as a consequence of natural enemy escape [29]. If dispersal is adaptive, then perhaps there are varying circumstances under which the probabilities of dispersal and distances moved tend to rise.

Spruce budworm and forest tent caterpillar are the most serious forest insect pests on conifers and broad-leaved trees, respectively, in Canada’s boreal forest. In both cases, dispersal is thought to play a critical role in triggering localized population eruptions and synchronizing large-scale outbreak cycles, although it is also clear that forest landscape structure serves to limit the degree of spatial synchrony in outbreak cycling in both spruce budworm [30] and forest tent caterpillar [31]. Is it through the effect of forest landscape structure on dispersal that it controls the degree of spatial synchrony in outbreak dynamics?

We now have evidence that adult female dispersal is density-dependent in the case of spruce budworm [32]; however, no such evidence currently exists for forest tent caterpillar. If dispersal in these pests is density-dependent, is it also dependent on spatial variations in host forest landscape structure? Under an adaptation hypothesis, one might expect adult females to be repelled by high density of conspecifics, and attracted to higher densities of high-quality host trees, which would in turn lead to more intense and better synchronized outbreaks in forests rich in host species.

In this paper, I present field data from two independently designed population studies, both initiated in Alberta, Canada in the mid-1990s, examining the effect of forest landscape structure on adult female movement in forest tent caterpillar and spruce budworm. The two species merit deep consideration because they serve as model systems for comparison with other taxa. In fact, these two genera, *Choristoneura* and *Malacosoma*, represent clades of 10 and 3 species in North America, and 46 and 14 species worldwide. If dispersal can be shown to be adaptive in these two model species systems, then perhaps the inference extends across the genera, and even Lepidoptera as whole.

## 2. Materials and Methods

### 2.1. Forest Tent Caterpillar

The forest tent caterpillar study at Cooking Lake, Alberta (50 km East of Edmonton) was conducted on a 20 km × 15 km (30,000 ha) grid of 61 population sample points, over a heterogeneous forest landscape structure dominated by trembling aspen (*Populus tremuloides* Michx.) host trees and mixed agriculture. The landscape and its long-term history of forest tent caterpillar outbreaks and population dynamics has been described in detail elsewhere [28,33,34,35]. This includes the sampling methods by which moth densities and egg mass densities were made, and the methods for estimating forest cover and defoliation levels. Forest cover, measured as percent forest, based on digital aerial photography taken in 1993, was estimated over circular neighbourhoods in a geometric series of diameters ranging from 53 m to 1700 m, in order to determine the scale at which insects might be responding to forest landscape structure. Defoliation was estimated from the ground as percentage of crown foliage removed.

Egg mass densities were computed as egg masses per tree crown. These egg masses were collected in the summer of 1996, from trees that had fallen down in the spring of 1996, as described in [33]. Having full access to large tree crowns allowed us to do a complete census of crown populations, which is normally infeasible. Old egg masses are retained on twigs in the crown for a number of years, but eventually fall off, due to twig radial growth and weathering. Thousands of old egg masses were collected, and these were classified as to the year of origin, based on the degree of weathering. In this study, I focused on the two years—1994 and 1995—for which there were egg mass density estimates at enough sites to evaluate the likely movement patterns of gravid females. In theory, I could have analyzed egg mass densities from 1993 as well as 1994 and 1995; however confidence in aging accuracy and sample size both tend to decrease the older the egg mass and the heavier the weathering.

Moth densities were estimated as the density of non-parasitized cocoons in a timed collection (cocoons per minute) made from the shrub layer in late June of the specified year. Sex ratios were not available for this study population, so the density of all moths was used, rather than just the female component. The sex ratio is typically 1:1 in forest tent caterpillar, and does not deviate to unbalanced as in some other species. Our use of adult density in place of female density implicitly assumes the sex ratio is relatively constant across the study area.

I used the method of Royama [36] to calculate the ratio of eggs (E) to female moths (M)—or “E/M ratio”—as an index of net migration, although I used egg mass density (egg masses per tree crown) in place of egg density, and adult density (adult moths per minute) in place of female density. The biases resulting from these substitutions does not compromise the analysis, however, as the hypothesis was regarding spatial variation in E/M ratios, and not their absolute values.

A relatively high E/M ratio indicates a higher probability of net immigration of gravid females into a given plot. A relatively low E/M ratio indicates a higher probability of net emigration of gravid females out of a given plot. Spatial variations in E/M ratios, and hence immigration rates, could thus be related to spatial variations in independent predictors, such as defoliation levels or amount of forest cover. I predicted that gravid females in 1994, and 1995, at the peak of an outbreak cycle, would tend to move away from heavy defoliation and toward more dense forest cover. I used spatial regression analysis to determine if E/M ratios in 1994 and 1995 were related to defoliation levels and to forest cover. I included three geographic terms (Northing, Easting, Northing × Easting) to account for any large-scale systematic spatial advection that might obscure local responses to defoliation and forest cover.

### 2.2. Spruce Budworm

**Figure 1:**
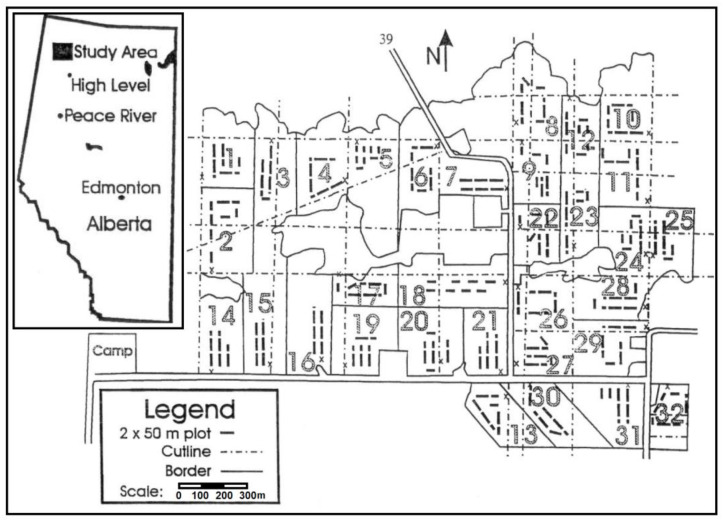
A schematic representation of the Zama silvicultural installation. 344 ha of treatment compartments are distributed randomly over an area roughly 2.5 km × 1.5 km (375 ha) in size.

The spruce budworm study was conducted on a 2.5 × 1.5 km (375 ha) matrix of forest blocks in boreal northwestern Alberta, near Zama City, in parts of Township 118 Range 3 and Township 119 Range 3, both west of the 6th principal meridian (59°20′ N, 118°27′ W) in the Forest Management Agreement managed by High Level Lumber Division, Tolko Industries Ltd. (Figure 1). The study area was 85% white spruce (*Picea glauca* (Moensch) Voss) and 15% trembling aspen. Excluded from the study design were 31 ha of wetland, making the total area sampled 344 ha.

The spruce budworm study began in 1997, just as the forest tent caterpillar outbreak at Cooking Lake was collapsing. The forest blocks consisted of various levels of partial harvest, as part of a long-term plan to investigate the effect of modified silviculture on spruce budworm outbreak dynamics [37]. Although the installation burned in the summer of 2012, before the experiment could be completed (final observations were scheduled for 2067), spatial population data from the years 1997–2011 could be used to examine E/M ratios over time as a function of forest cover.

**Figure 2:**
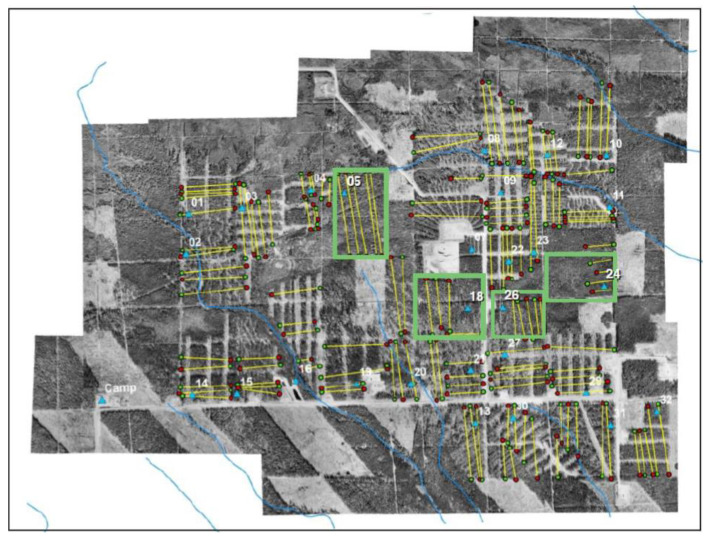
Aerial photograph of the Zama silvicultural installation, numbered compartments comprising 344 ha in area. The green outlined blocks are the unharvested “control” compartments: 05, 18, 24, 26. Yellow lines are sample transects used in population sampling.

Forest cover in the study area was made to vary according to a randomized block design, with three different levels of white spruce host tree removal rate (measured as a proportion): either 0.25 or 0.5 basal area removed, or 0.0 removal rate for uncut blocks. The partially harvested blocks were replicated 12 times, and the uncut blocks were replicated four times, yielding 28 sample plots over the 344 ha installation (Figure 2).

Between 1997 and 2011 inclusive, pheromone traps were used to catch adult males, and per branch egg mass densities were estimated by sampling a minimum of one branch per tree (mean = 1.18 branches per tree) on a minimum of 1–2 trees per block (mean = 3.12 trees per block), yielding a mean of 3.7 sample branches per block. Spruce budworm pheromone traps were deployed when most larvae had ceased feeding and pupation was underway. Two traps were placed in each compartment in the vicinity of the egg mass sample trees, for a total of 56 traps. These traps were collected when egg mass samples were obtained. The number of spruce budworm adults in each trap was counted by taking the average number of individuals that would fit into a small cup. The total number of individuals was calculated by multiplying the number of full cups by the average and adding this to the remaining number of individuals only filling a partial cup. To measure egg mass densities, a 12-gauge shotgun was used to remove individual branches from the mid-crown from previously selected sample trees. The width of each branch was measured and each branch was cut to a standard length of 45 cm. In the lab, all egg masses on each branch were tallied to express their densities as egg masses per m^2^ of foliage.

As in the forest tent caterpillar study, the “E/M ratio” was used as an indicator of net movement in or out of a population, except in this case I used actual egg mass densities per m^2^ of foliage, and not just egg mass counts per tree crown. When E/M is relatively high, there are more budworms in a sample than one could expect based on a normal fecundity of roughly 200 eggs per female moth, implying that the extra eggs must have come from immigrant females. When E/M is relatively low, it conversely implies that egg-bearing females must have emigrated from the area being sampled. E/M typically varies widely among years, and is the primary source of sawtooth fluctuations around the spruce budworm’s population cycle [38]. As stands become heavily defoliated through time, E/M tends to drop, as moths move out of declining stands and into more healthy stands [39].

Typically, the density of female moths is estimated by measuring pupal densities and then multiplying by the sex ratio of females in the sample. This was not possible in the spruce budworm study because pupal densities and sex ratios were not available for all blocks in all years. What was available as a measure of adult population density was male pheromone trap catch. If female and male moths were present in equal numbers, one could use male pheromone trap catches as a robust proxy for female moth densities. However—as in the forest tent caterpillar study—the matter of sex ratios may be set aside, as the primary interest was not in the absolute value of the E/M itself, but, rather, systematic patterns of change in E/M among treatment blocks. If distinct forest blocks have no influence on moth movement, then E/M should be constant across the study area, regardless exactly how E or M is estimated. All that matters is that any bias in the E/M ratio estimate is consistent across sample points.

The constancy of E/M ratios among years and forest treatment blocks was tested using ANOVA. All statistical analyses were conducted using R, version 4.1.3 [40].

## 3. Results

### 3.1. Forest Tent Caterpillar

**Figure 3:**
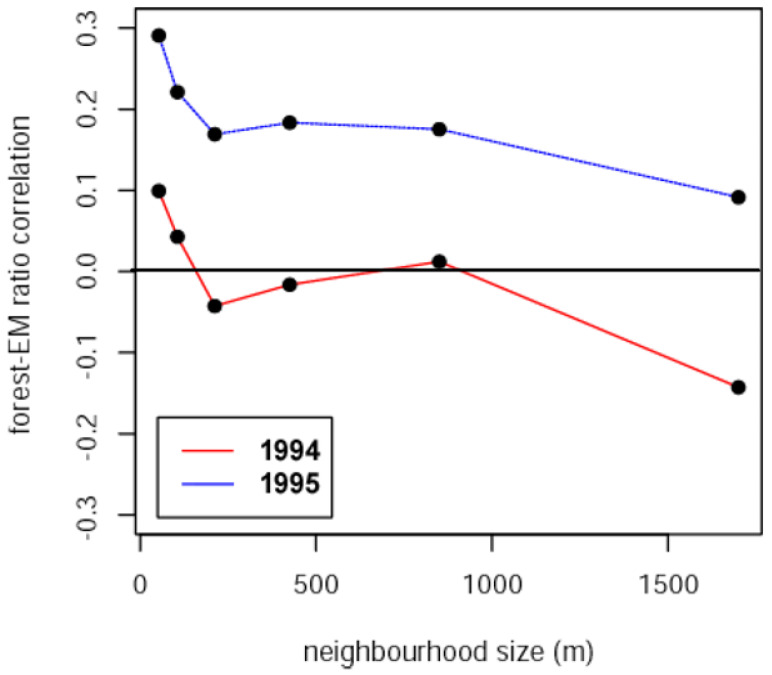
Correlation between forest tent caterpillar E/M ratios and forest cover measured over various neighbourhood sizes, from 53 m to 1700 m, for 1994 and 1995.

Forest tent caterpillar E/M ratios varied tremendously across the study area in both 1994 and 1995, from near zero to nearly three. E/M ratios were generally higher in 1995 than 1994, with the bulk of that difference coming from the 18 plots lining the southern limit of the area sampled, indicating a more southerly direction of migration in 1995. The correlation between E/M ratios and forest cover in both years was maximal at the shortest distance class of 53 m, and this correlation was higher (across all distance classes) in 1995, the same year when E/M was higher (Figure 3).

**Figure 4:**
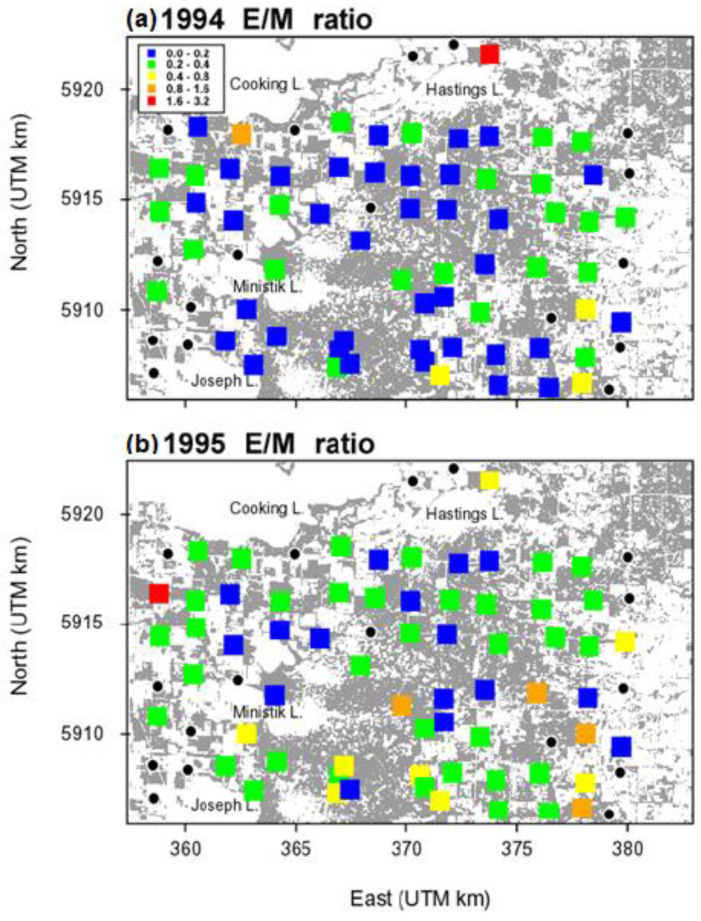
The spatial pattern of forest tent caterpillar E/M ratio across the 20 km × 15 km Cooking Lake study area, in (**a**) 1994 and (**b**) 1995. Grey shading indicates forest cover, primarily trembling aspen. Black circles are population plots for which the egg density or moth density was missing.

The correlation between E/M and forest cover in 1994 switched from weakly positive at the shortest distance class (53 m) to weakly negative at the largest distance class (1700 m), consistent with the interpretation of a weak trend toward adult females moving out of the central part of the more heavily forested part of the landscape where the outbreak was centred, and radially outward, toward the eastern and western edges where the landscape was more fragmented (Figure 4).

Regression of E/M ratios on rates of defoliation and forest cover measured at the largest and smallest distance classes indicated strong effects of both variables in both years, even after controlling for geographic trends (Table 1). These effects were similar in sign among years, varying only in magnitude and significance level. In 1994, the negative effect of defoliation was more significant than the positive effect of nearby forest (overall model R^2^ = 0.43). In 1995, there was a geographic trend to the E/M pattern and the positive effect of nearby forest was more significant than the negative effect of defoliation (overall model R^2^ = 0.25). The intercepts differed significantly between years, indicating an overall difference in dispersal success, 1995 exhibiting higher levels of adult female movement.

### 3.2. Spruce Budworm

**Figure 5:**
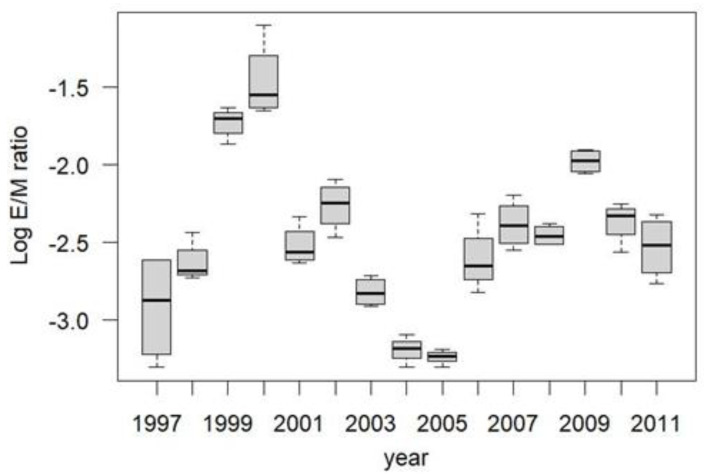
Zama spruce budworm Log_10_ E/M ratios, 1997–2011. Years 1999, 2000, 2009 indicate anomalously high E/M ratios that may have been the result of mass immigration from outside the study area. (Log transformation was used to stabilize variances among years).

The spruce budworm outbreak at Zama came in two distinct waves of high egg recruitment: 1998–2003 inclusive and 2006–2011 inclusive, with E/M ratios varying tremendously among years, and peaking in 1999–2000 and 2009 (Figure 5).

**Figure 6:**
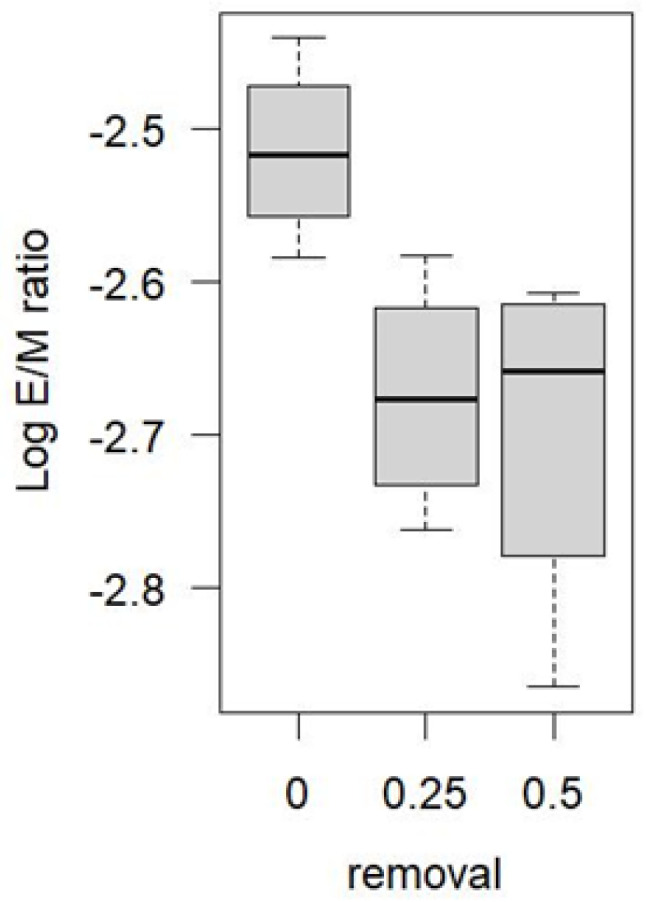
Zama spruce budworm Log_10_ E/M ratios, by removal rate, measured as proportion of forest removed (0 signifies unharvested blocks). Years of potential heavy immigration (1999, 2000, 2009) were removed from analysis. (Log transformation was used to stabilize variances among years.).

In order to examine the potential effect of local harvest treatment on local moth movement patterns, I excluded the three anomalously high years of recruitment—1999, 2000, and 2009—and examined the E/M ratios across all blocks in all other years. E/M ratios in the unharvested blocks were significantly higher than in the partially harvested blocks (Figure 6).

An analysis of variance suggested that once the extreme variability in E/M ratios between years was accounted for, the negative effect of partial harvest on recruitment in Figure 6 was significant (Table 2)—an effect that was not significant when the years 1999, 2000, and 2009 were included in the analysis.

## 4. Discussion

The forest tent caterpillar outbreak at Cooking Lake that peaked in 1993 was followed by two years of significant expansion and redistribution of female moths and their progeny, as reflected in spatial patterns in E/M ratios, in both 1994 and 1995. Moths were repelled by higher defoliation in the centre of the study area in both years and attracted to areas around the perimeter that were less defoliated and had more finely scaled forest cover. As the outbreak subsided due to low E/M ratios in the centre of the outbreak core, moths were moving further afield for better forest conditions: sites with lower defoliation that were not as extensively forested as the core area, but nevertheless well-forested at fine scales. The net flow was both East and West in 1994 (which is why the linear geographic terms were non-significant in 1994), and South in 1995. It is noteworthy that it was forest cover locally (measured at 53 m), and not in the broader neighbourhood (1700 m), that was exerting the positive influence on egg recruitment (Table 1). With such a highly pseudoreplicated design it is always possible that forest cover is merely acting as a proxy for the radial diffusion of moths outward from the core outbreak area.

The spruce budworm outbreak at Zama started in 1996 but accelerated with anomalously high E/M ratios in 1999 and 2000. E/M ratios were highly variable at all time and space scales, but the high degree of replication allowed me to show that E/M ratios were consistently higher in the unharvested blocks than in the partially harvested blocks. Excluding all years of suspected immigration from afar (1999, 2000, 2009) suggested the effect was significant: egg-bearing females were less likely to emigrate from the unharvested blocks, and/or more likely to immigrate to them.

A study of spatial variation in E/M ratios cannot resolve whether areas of low recruitment serve as population sources for areas of high recruitment. However, a review of the study area design (Figure 2) helps to clarify this possibility. Although the treatment blocks in the original design were assigned randomly, the unharvested blocks turned out to occupy a central part of the study area, and were clustered together somewhat, with three of the unharvested blocks actually neighbouring one another (18, 24, 26), and with the fourth (05) just 200 m from them. Given the arrangement of the blocks, it is plausible that there was a collective flow of females from the partially harvested periphery of the study area toward the unharvested center.

In both studies, there was clear affinity of gravid females for more heavily forested areas, but the spatial patterns of net migratory flow were opposite among species. In the forest tent caterpillar study, the female moths were repelled by defoliation in the core outbreak area and were attracted to locally dense aspen forest cover on the periphery. In the spruce budworm study, the female moths were more likely to move from the partially harvested peripheral blocks into the unharvested core of dense spruce cover. It is not surprising, perhaps, that defoliators should move away from defoliation and toward more abundant host cover. On the other hand, this is a rare case where empirical evidence was obtained in support of this common assumption. This demonstrates that local dispersal by female moths of both species is adaptive.

A significant limitation in both studies is that no attempt was made to evaluate the role of weather in the flight period in promoting dispersal. Furthermore, these studies were not designed to address what might have happened to long-range dispersers, i.e., forest tent caterpillar flying distances greater than ~15–20 km, or spruce budworm flying greater than ~2 km, and thus out of the study area. Long-range dispersal is an important question because it may be responsible for the prolonging of outbreaks, as a result of temporary escape from natural enemies. The effects of weather and the nature of long-distance dispersal are worthy targets of additional research.

The context and scaling of the two studies differ greatly, and this requires comment, in order to make fair comparisons across studies. In the case of the 30,000 ha Cooking Lake study area, it is an island forest in the aspen parkland region, less than 60 km from the largest city in Alberta, with no possible sources of moth immigration within 100 km, and no forest tent caterpillar outbreak anywhere else in the province during those years. The Zama installation, in contrast, is one tiny parcel in a vast matrix of boreal forest, with spruce budworm outbreak occurring contemporaneously in the surrounding landscape. This is why the spruce budworm study needed to treat three years of heavy immigration (1999, 2000, and 2009) as outliers, in order to examine local movement dynamics in the other years. These contexts are radically different.

With respect to scaling, the forest tent caterpillar study used 61 evenly distributed population plots over 30,000 ha of natural landscape, while the spruce budworm study used 28 evenly distributed population plots over 344 ha of experimental design. Yet, all anecdotal accounts indicate that the stout-bodied forest tent caterpillar, which lays her eggs in a single mass, is a less willing disperser than the slender spruce budworm, which lays her eggs in numerous clutches. The studies were therefore carried out at radically different spatial scales, with different design concepts, which helps to understand why different analytical methods were used for the two designs: spatially explicit regression analysis for forest tent caterpillar, and spatially implicit ANOVA for spruce budworm. Notably, the two disparate studies converged on a singular result in spite of these significant differences in scaling, context, and methodology.

The difference in scaling matters functionally, because after 15 years, 1997–2011, the spruce budworm study over 344 ha was showing no differentiation in population trajectories among treatment blocks; all populations cycled with the same frequency and amplitude over the course of one cycle, regardless of harvest treatment. In contrast, forest tent caterpillar population trajectories over 30,000 ha diverged strongly among landscape cover types over the course of a single cycle, 1993–2004 [34]. In hindsight, it is possible that the Zama study was of such a small scale relative to the dispersal capacity of spruce budworm that no amount of observation time would have been sufficient to observe a differentiation of spatial population dynamics. Over such short distances, moth dispersal may eliminate all local stand effects on population growth, leading to complete population cycle synchronization. Scale and context appear to matter greatly in forest insect landscape ecology, and the forest tent caterpillar study, in retrospect, seems well-scaled compared to the spruce budworm study.

## 5. Conclusions

Forest tent caterpillar adult females are able to redistribute themselves efficiently at the scale of a 20 km × 15 km study grid. It should come as no surprise that high-density forest tent caterpillar populations are averse to defoliation and tend to move toward areas with higher percent forest. This is an adaptive strategy that helps to avoid the severely negative consequences of crowding, from starvation to disease to predation and parasitism. On the other hand, it is valuable to have evidence to support any model assumptions regarding the density-dependence of female moth dispersal.

The spruce budworm outbreak at Zama 1997–2011 came in two distinct waves and affected all blocks equally. This homogenization of population trajectories, which one expects under the powerful synchronizing effect of rampant dispersal, occurred despite year after year net flow of eggs from the partially harvested blocks into the unharvested residuals located near the centre of the study area. Additional population analyses would be required to explain why the constant flow of eggs into the non-harvested block did not result in faster growing populations or greater rates of defoliation there.

In retrospect, the idea of a randomized block design over a small study area was not useful for studying the local population dynamics of an insect such as spruce budworm that is moving in large numbers over areas much wider than a few kilometres. In contrast, the spatial regression approach used with the larger forest tent caterpillar study area was well-suited to a landscape that was essentially an island forest closed to mass immigration from afar. Other studies of other species can learn from this lesson, and should take into account the importance of both context and scaling relative to the species biology.

Adult female dispersal appears to be highly adaptive in these two species, optimized for detecting high-quality host forest conditions for the next generation. Indeed, the more robust dispersal ability of spruce budworm relative to forest tent caterpillar may be indicative of the genera at large, as the *Choristoneura* tend lay eggs in multiple clutches, and the *Malacosoma* tend to lay eggs in a single clutch. This ability to immigrate into a wider array of host species niches may, in turn, explain why *Choristoneura* is three times as speciose as *Malacosoma*, both in North America, and worldwide.

## Figures and Tables

**Table 1:** Results from linear regression of forest tent caterpillar E/M ratio onto defoliation, forest cover, and geographic trend variables, for the years 1994 and 1995.

1994	Estimate	Std. Error	t-Value	*p*-Value
intercept	2.765 × 10^3^	2.604 × 10^3^	1.06	0.29
1994 defoliation	−4.962 × 10^−3^	8.625 × 10^−4^	−5.75	<0.001
Forest @ 53 m	1.451 × 10^−2^	4.969 × 10^−3^	2.92	0.005
Forest @ 1700 m	2.903 × 10^−3^	8.999 × 10^−3^	0.32	0.748
Elevation	−1.751 × 10^−4^	7.845 × 10^−4^	−0.22	0.82
East	−7.635 × 10^−3^	7.022 × 10^−3^	−1.09	0.28
North	−4.681 × 10^−3^	4.404 × 10^−4^	−1.06	0.29
East × North	1.293 × 10^−9^	1.187 × 10^−9^	−1.09	0.28
1995				
intercept	−6.178 × 10^3^	2.866 × 10^3^	−2. 14	0.037
1995 defoliation	−3.467 × 10^−3^	1.081 × 10^−3^	−3.21	0.002
Forest @ 53 m	2.024 × 10^−2^	5.375 × 10^−3^	3.77	<0.001
Forest @ 1700 m	5.260 × 10^−5^	9.604 × 10^−3^	0.005	0.99
Elevation	6.341 × 10^−4^	8.630 × 10^−4^	−0.73	0.46
East	1.684 × 10^−2^	7.779 × 10^−3^	2.16	0.034
North	1.045 × 10^−3^	4.880 × 10^−4^	2.14	0.037
East × North	−2.847 × 10^−9^	1.316 × 10^−9^	−2.16	0.034

**Table 2:** Results from ANOVA of spruce budworm E/M ratio 1997–2011, against year and forest removal rate, excluding years of potentially heavy immigration from afar: 1999, 2000, and 2009.

	Estimate	Std. Error	t-Value	*p*-Value
intercept	−95.4	26.0	−3.67	<0.001
year	0.046	0.013	3.57	<0.001
removal	158.6	71.09	2.23	0.0263
year × removal	−0.079	0.035	−2.23	0.0261

## Data Availability

Data are archived at https://doi.org/10.5061/dryad.r7sqv9sgm accessed on 17 October 2022.
